# Older Adults with Mild Cognitive Impairments Show Less Driving Errors after a Multiple Sessions Simulator Training Program but Do Not Exhibit Long Term Retention

**DOI:** 10.3389/fnhum.2016.00653

**Published:** 2016-12-27

**Authors:** Normand Teasdale, Martin Simoneau, Lisa Hudon, Mathieu Germain Robitaille, Thierry Moszkowicz, Denis Laurendeau, Louis Bherer, Simon Duchesne, Carol Hudon

**Affiliations:** ^1^Department of Kinesiology, Faculty of Medicine, Université LavalQuebec City, QC, Canada; ^2^Centre intégré universitaire de santé et de services sociaux de la Capitale-Nationale et Centre d’excellence sur le vieillissement de QuébecQuebec City, QC, Canada; ^3^Computer Vision and Systems Laboratory, Department of Electrical Engineering, Université LavalQuebec City, QC, Canada; ^4^PERFORM Centre, Concordia UniversityMontreal, QC, Canada; ^5^Department of Medicine, University of Montreal and Montreal Heart InstituteMontreal, QC, Canada; ^6^Centre de recherche de l’Institut universitaire en santé mentale de QuébecQuebec City, QC, Canada; ^7^Département de Radiologie, Faculté de Médecine, Université LavalQuebec City, QC, Canada; ^8^École de psychologie, Université LavalQuebec City, QC, Canada

**Keywords:** MCI, driving, learning, training, retention

## Abstract

The driving performance of individuals with mild cognitive impairment (MCI) is suboptimal when compared to healthy older adults. It is expected that the driving will worsen with the progression of the cognitive decline and thus, whether or not these individuals should continue to drive is a matter of debate. The aim of the study was to provide support to the claim that individuals with MCI can benefit from a training program and improve their overall driving performance in a driving simulator. Fifteen older drivers with MCI participated in five training sessions in a simulator (over a 21-day period) and in a 6-month recall session. During training, they received automated auditory feedback on their performance when an error was noted about various maneuvers known to be suboptimal in MCI individuals (for instance, weaving, omitting to indicate a lane change, to verify a blind spot, or to engage in a visual search before crossing an intersection). The number of errors was compiled for eight different maneuvers for all sessions. For the initial five sessions, a gradual and significant decrease in the number of errors was observed, indicating learning and safer driving. The level of performance, however, was not maintained at the 6-month recall session. Nevertheless, the initial learning observed opens up possibilities to undertake more regular interventions to maintain driving skills and safe driving in MCI individuals.

## Introduction

Mild cognitive impairment (MCI) is characterized by objective memory impairments with or without other cognitive deficits such as language, attention or executive function disorders (Petersen, 2004). The autonomy of many MCI individuals is relatively preserved, but a gradual functional decline can be observed when the cause of this syndrome is a neurodegenerative disease. One of the most frequent causes of MCI is Alzheimer’s disease (AD; Elias et al., 2000; Albert et al., 2011). Although there is some variability between studies regarding its prevalence, MCI could affect between 3% to 32% of the elderly population (65 years and older; Ward et al., 2012).

Compared to older adults with normal cognition, individuals with MCI and those with clinical mild dementia show similar rates of driving cessation and frequency (O’Connor et al., 2010). Also, there are several questionnaire studies showing that a significant proportion of older individuals with a diagnosis of MCI or dementia are active drivers and will continue to drive for several years after having received their clinical diagnosis (Silverstein, 2008; Betz and Lowenstein, 2010; Turcotte, 2012; O’Connor et al., 2013). Although it is well known that older drivers tend to underestimate the number of trips they take and provide inaccurate estimates of their traveled distance (Blanchard et al., 2010; Porter et al., 2015), these data are important because they suggest that older individuals with MCI are active drivers.

So far, all studies that have examined driving among MCI individuals reported that most of these persons can drive safely (Wadley et al., 2009; Frittelli et al., 2009). However, maneuvers involving executive functions, such as left turns (i.e., crossing a lane with traffic going the opposite direction), changing lanes and maintaining the vehicle in the center of the lane, are considered suboptimal in participants with MCI (Grace et al., 2005; Dawson et al., 2009; Wadley et al., 2009). In some driving simulation studies, drivers with MCI have shown poor control of speed (driving at slow speed and greater variability of speed than healthy individuals) and lateral position (weaving, driving off the road), and improper distance with a lead vehicle (Freund et al., 2002; Pavlou et al., 2016).

As cognitive function declines and dementia progresses, driving can become a serious traffic safety problem (Hunt et al., 1993, 1997; Dubinsky et al., 2000; Rizzo et al., 2001; Uc et al., 2005). Because the driving abilities of individuals with MCI are expected to worsen with the decline of cognitive capabilities, there is currently a debate about whether or not individuals diagnosed with MCI should also be allowed to continue to drive (Olsen et al., 2014). However, a recent Cochrane review (Martin et al., 2013) clearly highlights the lack of construct validity of current approaches to assess driving performance and the identification of at-risk drivers. Using data from a large-scale prospective cohort study, the Maryland Prospective Older Driver Study (Staplin et al., 2003), the authors have estimated that the cognitive test that most strongly predicted future crashes would, if used as a screening tool, potentially prevent six crashes per 1000 people over 65 years of age screened. This, however, would be achieved at the price of stopping the driving of 121 people who would not have had a crash. Martin et al. (2013) suggested that, although declining driving skills raise understandable concerns about crash risk, these data suggest that screening currently discriminates unfairly against older drivers. A similar suggestion arises from the work of Jeong et al. (2012) who showed no differences in the history of crashes and traffic citations for a period of 3 years between healthy elderly drivers and older drivers with MCI.

Clearly, the identification of at-risk drivers is problematic (Bédard et al., 2008; Gamache et al., 2010; Bédard and Dickerson, 2014). For older drivers with MCI, transitioning to alternative transportations is an option that needs to be considered as driving cessation will occur in the future (Carr and Ott, 2010; Wheatley et al., 2014). Before this potential transition occurs, one approach could be to examine whether or not these individuals can be retrained to maintain or even improve their actual level of driving performance. An interesting observation is that procedural memory (implicit learning) is preserved in individuals with MCI, as well as in people having clinically probable AD (McEvoy and Patterson, 1986; van Halteren-van Tilborg et al., 2007; Gobel et al., 2013). Implicit learning, contrary to explicit learning, takes place without awareness, often by repetition, and without reference to explicit knowledge learned previously (Vinter and Perruchet, 2002; van Halteren-van Tilborg et al., 2007). According to Willingham (1998) and Willingham and Goedert-Eschmann (1999), there could be an interaction effect between implicit and explicit learning, and implicit motor-skill learning could take place in parallel during explicit learning when a movement response is done. Complex sequence of actions can be learned implicitly (Witt and Willingham, 2006). With driving, knowledge of road safety rules is explicit knowledge, but several of the maneuvers involve implicit learning. For example, people explicitly know they should brake at an intersection with a stop sign, but releasing the accelerator and dosing the pressure on the brake pedal when braking is an implicit learning task that takes place while practicing. With healthy individuals, several studies have reported the maintenance of the acquired skills several months after implicit learning (Albouy et al., 2008; Gheysen et al., 2010; Doyon et al., 2011; Rose et al., 2011; Simon et al., 2012). Furthermore, two recent studies have demonstrated that this type of intervention brings measurable functional changes in the brain in healthy adults (Oosterman et al., 2008; Gheysen et al., 2010).

There is currently a lack of evidence as to whether or not MCI patients can benefit from a driving training program. In a recent pilot study (Teasdale et al., 2016), we examined if individuals with MCI could benefit from a training program in a driving simulator (five sessions over a 3-week period). None of these individuals participated in the current study. For several maneuvers (speeding, not using the turn signal, verification of the blind spot, tailgating), a gradual and significant decrease in the number of errors was noted. Individuals with MCI also showed implicit learning, with their braking showing a shorter and more uniform deceleration with training. These data are important as they suggest that individuals with MCI can be trained to drive more safely. In this new research project, we wanted to replicate these initial results. As well, we had a particular interest in testing if this initial learning decays when there is no rehearsal. To test this hypothesis, we recruited a group of drivers with MCI. They first participated in a 5-session training program within a 21-day period. Then, a 6-month recall session without any feedback was given. This last session was a transfer test serving the purpose to examine if the participants were able to transfer the improved performance observed within the first 21 days (5 sessions) to a new context approximating what is needed in a real-world setting (i.e., driving alone without any additional verbal feedback on the performance; Lee, 1988; Schmidt and Bjork, 1992).

## Materials and Methods

### Participants

Fifteen elderly individuals with amnestic MCI (eight single-domain; seven multiple-domain) were recruited from memory clinics. Participants had a valid driving license, normal or corrected to normal vision (6/15 or better on the Snellen test) and declared driving regularly (>3 times a week). This experimental group included 13 men and 2 women (age range: 60–89, mean age (±SD): 72.0 ± 8.8 years, education (years ± SD): 14.6 ± 2.7). None of the participants had a significant decrease in functional autonomy, but all were showing objective cognitive problems, including at least memory impairment. This decline in cognitive functioning was first detected through participants’ complaints, which in turn were confirmed by a close relative. To confirm the presence of MCI, procedures similar to those adopted by Gaudreau et al. (2015) were followed. Briefly, the MCI was confirmed based on a battery of clinical and neuropsychological tests that was administered. As well, each case was discussed by a team of clinicians in order to reach a consensus regarding the status of participants. All participants met the core clinical criteria for MCI as defined by Albert et al. (2011). On their first visit to the laboratory, participants were briefed about the requirements of the experiment and invited to read and sign an informed consent declaration approved by the Ethics Committee of the *Institut Universitaire en Santé Mentale de Québec*.

### Questionnaire and General Driving Assessment

All participants completed a general verbal questionnaire (driving habit questionnaire, DHQ) that included items on driving (frequency of driving and average km/week, presence of an accident during the last years; Owsley et al., 1999). This information regarding self-reports of driving exposure was only used to verify if participants were active drivers. As well, the DHQ includes several questions about avoidance behaviors during the past 3 months (driving outside the immediate neighborhood, left turns (crossing a lane with traffic going the opposite direction), night driving, bad weather, rush hours, highways). For each of these questions, there was also a secondary question regarding the confidence in their driving ability (5-point scale from 0 = no difficulty to 5 = great difficulty). A summary of the responses is provided in Table 1.

**Table 1:** **Summary of the self-reported driving habits**.

	Mean (standard deviation)	
Driving days per week	5.6 (1.3)	
Average km/week	216 (191)	
Presence of accident during the last years	1 MCI with two accidents, and 1 MCI with one accident	
During the last 3 months, did you avoid… (number of drivers)

Driving outside immediate neighborhood		0
Left turns (i.e., crossing a lane with traffic going the opposite direction		1
Night driving		4
Bad weather (rain)		0
Rush hours		1
Highways		0

Before each session, participants were asked if they were in their usual state of fitness (that is, not suffering from a cold or flu or hangover) and were made aware the simulator could make them feel uncomfortable (nausea, dizziness, general discomfort and headache). They were instructed to inform the experimenter if this happened and were told to stop the simulation session before they felt discomfort or illness that could lead to emetic responses. They were told the experiment would stop immediately without any prejudice for them. To prevent simulation sickness situations to occur, the temperature within the room was maintained around 19°C with proper airflow using a ceiling vent positioned just above the driver.

### Simulator

A fixed-based open-cab simulator powered by STISIM Drive 3.0 (System Technology Inc., Hawthorne, CA, USA) was used for training purposes. Images were projected on a screen (1.45 m high × 2.0 m wide) located 2.2 m from the steering wheel using a projector (Hitachi CPX8) displaying a 40° horizontal by 30° vertical field-of-view with the center of the screen located at eye-level through the mid-line of the subject. The simulator has an automatic transmission. Steering movements and displacements of the accelerator and brake pedals were also recorded (Computer Measurement PCI DAS08, 12-bit A/D) during driving. The simulator included a digital input/output board (Computer Measurement PCI-DIO24) allowing to record activation and deactivation of the turn signals. The cabin had genuine vehicle parts, and a fully instrumented dashboard (Tessier et al., 2009) leaving the entire screen for presenting the road environment. Audio feedback of the engine noise was provided through two speakers positioned in front of the vehicle. Two USB video cameras (Webcam C905, Logitech, Silicon Valley, CA, USA) were used, one was mounted on the cab facing the subject and zoomed to capture head and eye movements while the other one captured the scenario displayed on the screen. A magnetic tracker (Flock of Birds, Ascension Technology Corporation, Burlington, VT, USA) secured on the driver’s head recorded head movements while driving. To comply with the 40° field of view of our simulator, there was no right or left-turn maneuver at intersections (i.e., crossing a lane at a right angle with traffic going the opposite direction), and moderate curves were presented (smallest radius of curvature of 120 m).

To detect driving errors, custom-made software was developed using STISIM 3 open module. The open module fed all information about the scenario and the simulation to a second computer through an Ethernet TCP/IP connection. This information was processed in real-time to evaluate the driving performance. The software also included a module to determine head and eye movements when a lane change was performed (Metari et al., 2013). A description of the driving feedback that were provided is presented below.

### Procedure

At the first driving session, participants were explained the study, completed the DHQ, and were familiarized with the simulator. Then, they were given five simulator sessions on five different days within a 21-day period. A 6-month recall session was then held without any feedback. At each session, the participants drove a 6 km practice run (with less graphical information than the experimental scenario) to familiarize themselves with the simulator and recorded instructions. They were asked to comply with local traffic regulations throughout the experiment. The width and markings of the lanes were implemented according to governmental rules and speed limits, and advisory signs appeared throughout the scenario. Intersections with a stop sign or a traffic light were presented. No emergency braking response was necessary unless a driving error was made. During the familiarization run (for the first session as well as for all training sessions), general explanations were provided whenever a driver requested specific information regarding the auditory feedback that were provided, and the experimenter made sure that drivers understood the message relevant to each feedback and responded with appropriate corrective responses. The feedback provided were developed based on previous reports of typical errors reported for drivers having cognitive problems (Grace et al., 2005; Dawson et al., 2009; Wadley et al., 2009; Pavlou et al., 2016; Teasdale et al., 2016). They included maneuvers involving executive functions, such as changing lanes (indicating the intent to change lanes and verifying the blind spot) and proper control of the vehicle (speed, variability of the lateral position, and control of the vehicle at intersections). After the familiarization, participants rested for 5 min before they were given a continuous 27.48-km long scenario with urban and semi-urban two-way and four-way roads with minor grade changes. The scenario included recorded instructions to inform the driver about requested maneuvers (for example, instructions to overtake securely a slower-moving vehicle ahead of them) and conditional feedback about specific maneuvers when a driving error was detected by the simulator. No additional information was provided. For the 6-month recall session, the participants drove the familiarization session followed by the 27.48-km long scenario. As mentioned above, no feedback was given during this recall session. A brief description of the feedback provided is now presented.

#### Speeding

The scenario included urban and semi-urban sections with speed limits set at 35 km/h, 50 km/h and 70 km/h. The distance traveled within each of these zones was 650 m, 12,570 m and 14,260 m, respectively. Subsections were arranged within the scenario to represent naturalistic driving conditions. Throughout the drive, a threshold of 10-km/h above the speed limit was accepted (the actual speed was always available through an analog speedometer located within the simulator dashboard). Consequently, for each speed zone, exceeding the speed limit by more than 10 km/h triggered an immediate auditory feedback (“Your current speed exceeds the speed limit. You should slow down”). The driver had to reduce their speed below the 10 km/h threshold within the following 10 s to avoid an additional warning for the same speeding event.

#### Tailgating

This consists of driving too close to a frontward vehicle at a distance which does not guarantee avoiding a collision if stopping is required. The threshold was adapted to the speed of the driver using a time to contact measure. The threshold was set at 2 s. For example, at 50 km/h the minimum distance from the frontward vehicle needed to be greater than 27.7 m. A shorter distance would trigger a feedback (“Keep a safe distance from the vehicle preceding you”). Reducing the speed and/or increasing the distance from the frontward vehicle to increase the time to contact above the 2-s threshold within the next 10 s prevented the driver from receiving an additional feedback for the same tailgating event.

#### Weaving

Failure to control the lateral position of the vehicle is defined as weaving. In this study, we identified difficulties of drivers in maintaining the vehicle within the center of the road. A lateral positioning error was defined as maintaining the vehicle farther than 17.5% of the lane width from the center of the lane for 10 s. In other words, if the tires were less than 15 cm from the nearest road line for more than 10 s, a feedback was given (“You should maintain your vehicle in the center of the road”).

#### Lane Changing

Fifteen lane change maneuvers were included within the scenario. Some of those were requested through a recorded command indicating to overtake a slower vehicle safely and to move back into the rightmost lane. Others were integrated within the scenario through the road design (for instance, lanes that were merging). A few additional lane change maneuvers could occur as a function of the driver’s strategies. Whenever a lane change occurred, the system would verify the driver had signaled their intention to change lane before changing lanes (i.e., activation of the correct turn signal) and that a blind spot verification had occurred prior to initiating the lane change. When this was not the case, a feedback (“Verify your blind spot before changing lanes”, “Activate your turn signal before changing lanes” or “Activate your turn signal and verify your blind spot before changing lanes”) was provided as soon as the mid-line of the vehicle crossed the line separating two roadway lanes.

#### Vehicle Control at Intersections with a Stop Sign

Failure to stop completely at an intersection (speed <1 km/h for at least 1 s) or stopping beyond the stop line triggered a feedback as soon as drivers crossed the intersection (“You should stop your vehicle properly at the intersection”).

#### Visual Search at Intersections with a Stop Sign

Drivers were instructed to look ahead and on their left and their right side to verify clearance before they entered the intersection. Ignoring this visual search triggered a feedback as soon as drivers crossed the intersection (“Just before entering the intersection, look left, ahead and right to check that the way is clear”).

#### Vehicle Control at Red-Light Intersections

A permissive yellow-rule was adopted. Specifically, the driver could enter the intersection during the entire yellow interval, but a feedback was provided if the light turned red before the vehicle crossed the midpoint of the intersection (“You should stop at the intersection whenever the light is red”). As well, stopping beyond the stop line triggered a feedback as soon as drivers crossed the line (“You should stop your vehicle properly at the intersection”).

Whenever a feedback was provided, an automatic 7 s delay was imposed before any other feedback could be given. A driving error occurring within this period would not trigger a delayed feedback but the error was recorded. This prevented consecutive feedback that could potentially overload the driver. No other feedback was provided. As well, no account of the driver’s performance was given at the end of a session or before any given session. For the 6-month recall session, feedback was turned off and participants were simply asked to drive the same 27.48-km long scenario as safely as they would normally drive.

### Data Analysis

For each session, we first analyzed the duration for driving the scenario. The results were submitted to a one-way ANOVA (first five sessions). We then compiled the number of errors made by each driver for each of the eight different types of driving errors (speeding, tailgating, weaving, omitting to indicate lane change, omitting to verify a blind spot, failure to stop properly at an intersection with a stop sign, failure to engage a visual search before entering an intersection with a stop sign, failure to stop properly at an intersection with a traffic light).

Because of the small number of subjects included in this study and the distribution of the error data, nonparametric statistical tests were adopted. First, we tested for each variable if learning occurred across the five training sessions using a non-parametric Friedman one-way analysis of variance (ANOVA). Significant differences were further examined using a Wilcoxon rank-sum test to examine more specifically if an improvement in the driving performance occurred between sessions 1 and 5. Further comparisons between two sessions were also made using the Wilcoxon rank-sum test (comparisons between sessions 6 and 5 or between sessions 6 and 1). All analyses were conducted using Statistica 13.0 (Dell Statsoft). The level of significance was set at 0.05.

## Results

### Characteristics of the Participants

Overall, four participants (two men and two women) elected to stop their participation due to simulator sickness during the first session. Data for these individuals are not included in the 15 participants included in this study. The 6-month transfer session included 13 participants as 2 participants declined to come back for personal reasons. Table 2 shows the sociodemographic and clinical/cognitive characteristics of the 15 participants. The sample included 2 women and 13 men. The mean age was 71 years and the mean education level was 14 years. No participant had clinical depression, but all had mild episodic memory impairment. The other cognitive functions were preserved at the group level, but as indicated in the “Materials and Methods” Section, some amnestic MCI subjects had additionally non-memory impairment. The most frequent non-memory impairment included language (i.e., naming or fluency) and/or executive deficits (i.e., inhibition). We compared participants with executive deficits with those without such deficits on the total number of errors observed at each session (Kolmogorov-Smirnov Two-Sample Tests). All comparisons were not significant and data for the 15 participants are presented thereafter.

**Table 2:** **Mean (standard deviation) sociodemographic and clinical characteristics of the participants**.

	Raw score (SD)	*Z* score (SD)	Percentile (SD)
*Sociodemographic characteristics*			
Age (years)	71.7 (9.0)	–	–
Sex (Male/Female)	13/2	–	–
Education (years)	14.3 (2.5)	–	–
*General cognitive functioning*			
MoCA (/30)	24.3 (2.5)	−0.7 (1.0)	–
DRS (/144)	136.1 (4.9)	−0.1 (0.9)	–
*Depressive symptoms*			
GDS (/30)	4.8 (3.8)	–	–
*Episodic memory*			
RL/RI mean free recall 1, 2 and 3 (/16)	6.3 (1.8)	−1.7 (0.5)	–
RL/RI mean delayed free recall (/16)	7.2 (2.5)	−2.5 (1.5)	–
RL/RI mean total recall 1, 2 and 3 (/16)	37.0 (7.3)	–	6 (5.6)
RL/RI mean total delayed recall (/16)	13.4 (2.3)	–	6 (5.0)
3-min recall ROCFT (/36)	12.9 (6.1)	−0.5 (2.5)	–
*Visuo-perception and visuo-construction*			
Copy score ROCFT (/36)	29.4 (5.1)	−1.2 (2.0)	–
Size-match subtest (BORB) (/30)	27.1 (2.2)	−0.1 (0.9)	–
*Information processing speed*			
Coding subtest (WAIS-III)	52.1 (14.1)	0.1 (0.9)	–
*Language and semantic memory*			
BNT total (/15)	12.9 (1.9)	−0.1 (1.3)	–
Phonemic fluency (T-N-P)	31.6 (7.3)	−0.8 (0.8)	–
Semantic fluency (animals)	15.5 (4.9)	−0.5 (1.0)	–
PPTT (/52)	49.9 (1.1)	–	–
*Executive functions*			
Stroop D-KEFS, inhibition (s)	75.4 (27.2)	0.0 (1.2)	–
Stroop D-KEFS, switching (s)	72.1 (21.8)	0.4 (0.9)	–
Stroop D-KEFS, inhibition (errors)	2.5 (3.7)	0.0 (1.0)	–
Stroop D-KEFS, switching (errors)	4.0 (4.3)	−0.2 (1.2)	–

*Note. Z scores and percentiles were obtained from age- and education-adjusted normative data. BNT, Boston Naming Test; BORB, Birmingham Object Recognition Battery; D-KEFS, Delis-Kaplan Executive Function System; DRS, Dementia Rating Scale; GDS, Geriatric Depression Scale; MoCA, Montreal Cognitive Assessment; PPTT, Pyramids and Palm Trees Test; RL/RI, Rappel libre/rappel indicé; ROCFT, Reu-Osterrieth Complex Figure Test; WAIS, Wechsler Adult Intelligence Scale*.

### Sessions 1 to 5: Training

**Figure 1:**
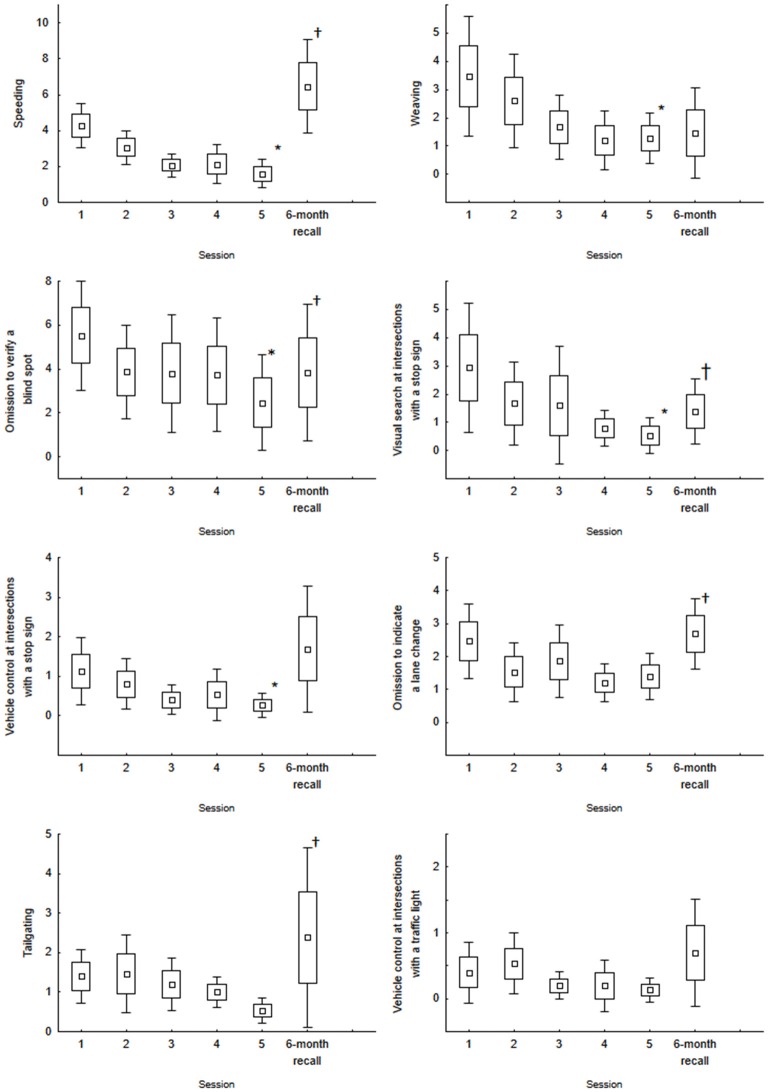
**Mean number of errors for the five training sessions and the 6-month recall session for speeding, weaving, omission to verify a blind spot, omission to engage visual search at intersections with a stop sign, vehicle control at intersections with a stop sign, omission to indicate a lane change, tailgating and vehicle control at intersections with a traffic light.** Box and Whisker indicate the standard error of the mean (±1.0 and ±1.96, respectively). *Indicates a main effect of Session (Session 1 to Session 5). ^†^Indicates a significant difference between Session 6 and Session 5. None of the comparisons between Session 6 and Session 1 were significant.

The time for driving the scenario did not vary across sessions. On average, driving the 27.48-km long scenario took 43 min, 10 s (*F*_(4,56)_ = 0.33, *p* = 0.855). The number of errors compiled for each session for each dependent variable is presented in Figure 1. Across the first five sessions, there is a general decrease in the number of errors for nearly all variables. The main effect of session was significant for speeding (*χ_(15,4)_* = 16.56, *p* = 0.002), weaving (*χ_(15,4)_* = 18.11, *p* = 0.001), omitting to verify a blind spot (*χ_(15,4)_* = 19.03, *p* = 0.0007), visual search at intersections with a stop sign (*χ_(15,4)_* = 24.39, *p* = 0.0000), and vehicle control at intersections with a stop sign (*χ_(15,4)_* = 10.16, *p* = 0.037). For this latter condition, we did not observe an intersection for which the driver did not stop. All errors were failure to stop completely at the intersection, or stopping too far from or beyond the stop line. The number of omissions to indicate a lane change was small (on average, 1.7 omissions per session for the five sessions), and the small decrease that was observed was not significant (*χ_(15,4)_* = 5.72, *p* = 0.22). Similar results were noted for the number of tailgating events (on average, 1.1 events per session; (*χ_(15,4)_* = 6.86, *p* = 0.143) and for vehicle control at intersections with a traffic light (*χ_(15,4)_* = 4.69, *p* = 0.319). As for intersections with a stop sign, we looked for major errors and noted two events where drivers stopped at a green light and two events where drivers did not stop at a red light.

### Session 6: 6-month Recall

Table 3 shows a summary of the comparisons between the 6-month recall session and the last training session (session 5) and between the 6-month recall session and the first training session. Mean values (and standard errors) are available in Figure 1. Overall, the analyses suggest a decrease in the performance between the 6-month recall session and the last training session (as expressed by a significant increased number of errors). This was observed for nearly all variables. The increased number of errors was significant for speeding, verification of the blind spot, omitting to engage in visual search before crossing an intersection with a stop sign, omitting to indicate a lane change, and tailgating. All other comparisons were not significant. Although we noted a decreased performance at the 6-month recall, when comparing the data with the first training session, none of the comparisons were significant.

**Table 3:** **Summary of the comparisons between the 6-month recall session and the first and last training sessions (Wilcoxon rank-sum tests)**.

	6-month recall vs. session 5		6-month recall vs. session 1	
Variable	*Z*	*p*-value	*Z*	*p*-value
Speeding	2.66	0.007	1.53	0.124
Weaving	0.15	0.878	1.92	0.054
Omission to verify a blind spot	2.03	0.042	0.57	0.563
Visual search at intersections with stop sign	2.2	0.027	0.11	0.905
Vehicle control at intersections with stop sign	1.12	0.262	0.17	0.858
Omission to indicate lane change	2.66	0.007	0.84	0.400
Tailgating	2.366	0.017	0.254	0.798
Vehicle control at intersections with a traffic light	1.21	0.225	0.314	0.753

**Figure 2:**
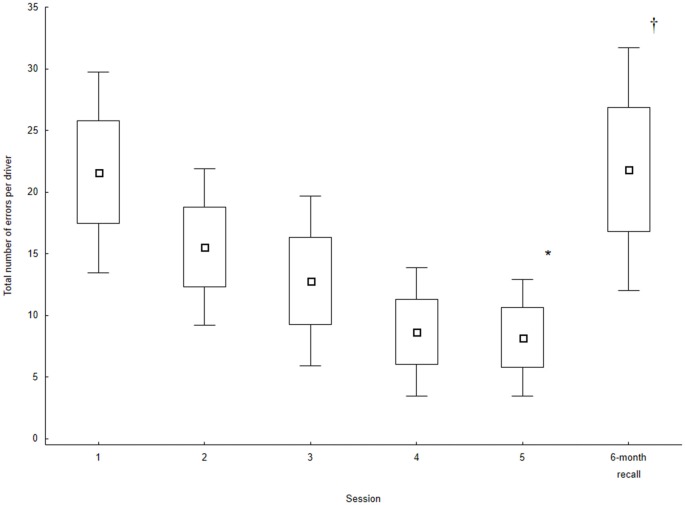
**Total number of errors (sum of all maneuvers evaluated) for the five training sessions and the 6-month recall session.** Values are the mean of all participants. Box and Whisker indicate the standard error of the mean (±1.0 and ±1.96, respectively). *Indicates a main effect of Session (Session 1 to Session 5). ^†^Indicates a significant difference between Session 6 and Session 5. The comparison between Session 6 and Session 1 was not significant.

Although, the number of errors observed for each variable could be considered small, the total number of errors is certainly not negligible. Figure 2 presents the mean number of errors per driver. For session 1, 21.6 errors were noted. This number decreased to 8.2 at session 5. The main effect of Session was significant (*χ_(15,4)_* = 39.36, *p* = 0.0001). The number of errors at the 6-month recall session increased to the level observed for Session 1 (21.8). The comparison between session 6 and session 5 was significant (*Z* = 3.17, *p* = 0.001) while that between session 6 and session 1 was not (*Z* = 1.13, *p* = 0.255). These errors were observed for a relatively short drive (27.48-km long scenario). Although few of these errors were critical (two for stopping at green-light intersections and two red-light crossings), the large numbers (at session 1 and at the recall) suggests that driving is not optimal in MCI individuals.

## Discussion

The main goal of this study was to examine if MCI individuals could benefit from a driving training program that provided automated real-time auditory feedback on various aspects of the driving performance known to be affected in drivers that are cognitively impaired (speeding, weaving, tailgating, omitting to indicate a lane change, omitting to verify a blind spot, vehicle control at intersections with a stop sign, omitting to engage in visual search before crossing maneuvers, crossing an intersection with a stop sign or a traffic light, and weaving). Also, an important objective was to determine if the benefits that could result from the initial training would be maintained at a 6-month recall session.

Overall, MCI individuals showed short-term improvements (five training sessions over a 21-day period). This was observed for speeding, weaving, omitting to verify a blind spot, vehicle control at intersections with a stop sign, and visual search at intersections. There was also a general trend for a decreased number of errors for the other variables that were analyzed (tailgating, omitting to indicate a lane change and vehicle control at red-light intersections). These results corroborate our previous observations also made with another group of MCI participants and a group of healthy older drivers (Teasdale et al., 2016). Contrary to a recent observation made by Pavlou et al. (2016), also in a simulator study, none of our participants drove with an excessively low speed and none maintained a large distance with the preceding vehicle. On the contrary, our participants exceeded the speed limit on several occasions (on average, four errors per driver for the first session with all drivers showing at least one speeding event; all but one driver showed an increased number of speeding events for the recall session) and several drivers also maintained a short time headway (tailgating; 11 out of 15 drivers showed at least one tailgating event on the first session). This was also observed in our previous study (Teasdale et al., 2016). Compared to the study of Pavlou et al. (2016), participants that were tested in our studies were at an earlier stage of MCI. Indeed, their participants included drivers with AD, Parkinson’s disease and MCI. Unfortunately, a lack of more specific information about how they diagnosed MCI makes direct comparisons difficult with their study. It may suggest, however, that as MCI progresses, more severe driving errors will come forth (Wheatley et al., 2014; Hird et al., 2016).

The validity of simulator studies is sometimes questioned. Simulator and on-road driving performance have been compared for different populations. These studies have confirmed the relative validity of driving simulators to assess on-road driving performance (Lee et al., 2003; Romoser and Fisher, 2009; Shechtman et al., 2009; Bédard et al., 2010; Mayhew et al., 2011; Lavallière et al., 2012). For example, Bédard et al. (2010) found a correlation of 0.74 between simulator demerit points and on-road demerit points in older drivers. More important, there are studies showing that training in a simulator allowed not only to improve driving in the simulator but also to transfer the learning to a better on-road driving performance. This was shown with healthy older drivers by Romoser and Fisher (2009) and Lavallière et al. (2012) and more recently, by Casutt et al. (2014). The training offered to older drivers in the studies by Romoser and Fisher (2009) and Lavallière et al. (2012) was individualized. Specifically, in Romoser and Fisher (2009), it emphasized visual scanning at intersections while in Lavallière et al. (2012), it emphasized lane change behaviors (indicating a lane change, verification of the mirrors and blind spot prior to changing lanes). In both studies, drivers who received a passive training (no feedback while driving in the simulator and a classroom-like training) showed no improvement in their driving performance. These results (beneficial effect of an active simulator training for improving on-road performance) were replicated by Casutt et al. (2014) in a study where the training consisted of increasing the mental workload by gradually increasing the traffic frequency, the number of virtual drivers ignoring traffic rules and hazardous traffic situations and by providing specific vigilance training. The key result from these three studies is that training improvements observed in a simulator transferred to an improved on-road performance. Furthermore, Lavallière et al. (2011) and Romoser (2013) both reported long lasting effects (2 years post-training) for the on-road performance for most drivers that participated in the active training. These studies are important because they clearly support the suggestion that active training in a simulator can benefit the on-road driving performance. In the current study, we showed clear improvements within the first five sessions and there is a likelihood this training also yielded to safer on-road driving, at least on a short-term basis. Future studies are needed to examine specifically if the improved simulator performance translates into safer on the road driving for MCI individuals.

An important feature of this study was the 6-month recall session. As mentioned in the “Introduction” Section, this last session served the purpose to examine if the participants were able to maintain their improved performance observed within the first five sessions to a new context approximating what is needed in a real-world setting (i.e., driving alone without any additional verbal feedback on the performance; Lee, 1988; Schmidt and Bjork, 1992). Our results show the improvements observed after the first five sessions were not long lasting as we observed a significant increase in the number of errors for four measures (speeding, omission to verify a blind spot, visual search at intersections with a stop sign, and omission to indicate a lane change). The number of tailgating events, which was small and did not vary significantly across the first five sessions, also increased significantly at the recall session. For instance, for speeding events, only one out of 13 participants maintained the level of performance observed at the fifth session (six for weaving, and seven for the omission to indicate a lane change). The performance at the 6-month recall, however, was not different than that observed at the first training session. Considering the large number of errors observed in session 1 and at the recall but the near absence of critical errors, this could indicate that MCI participants showed less than optimal performance. As mentioned in the “Introduction” Section, this fits the general description of the driving of MCI individuals both in simulator (Frittelli et al., 2009; Devlin et al., 2012), and on the road (Wadley et al., 2009). For instance, Devlin et al. (2012) reported an absence of significant differences when comparing the driving performance in a simulator of drivers with MCI to age-matched healthy drivers. In a previous simulator study, we also observed limited differences between MCI and healthy control drivers (Teasdale et al., 2016). The progression of MCI certainly could lead to a decreased performance. The present study opens up the possibility, however, that proper training could contribute to preserve and perhaps enhance driving competencies. This is an important result and future studies should aim at defining the optimal training conditions and regime for inducing safer driving in MCI individuals.

A large randomized, controlled clinical trial examining the long-term effectiveness of cognitive training on enhancing mental abilities (ACTIVE study, Unverzagt et al., 2009) showed that at a 2-year follow-up, MCI individuals did not benefit from interventions that were focused on declarative memory. Our study did not involve any training on issues such as trip planning/scheduling or navigation. These features of naturalistic driving clearly are associated with declarative memory (and with executive functioning) and individuals with MCI may experience more difficulties when driving involves these additional tasks. On the other hand, in the ACTIVE study, MCI individuals, just as healthy older adults, did benefit from training in reasoning and speed of processing, two qualities that are fundamental to driving. This is important as it indicates that future studies should consider establishing a list of driving skills that are pervious and impervious to training. Our study clearly shows, that several important driving skills can be improved in MCI individuals. This opens up possibilities to offer regular training to these individuals to maintain safe driving. At the same time, regular follow-ups may offer a window into the progression of cognitive decline and allow to better identify the limitations MCI drivers face.

The present study involved only 15 individuals with MCI. Although this is a limitation, it replicates previous findings observed for the first five sessions (Teasdale et al., 2016). More importantly, it shows that at a 6-month recall session, the MCI drivers were unable to maintain the level of performance they attained at the end of the training session (Figure 2). Our results also show large individual differences. These differences could be related to variability in the progression of cognitive decline and intrinsic differences in the driving style. Only three drivers showed a total number of errors smaller than 10 at the recall session. Two of these drivers exhibited safe driving throughout the training and at the recall (that is, less than 10 errors throughout all sessions). A third one who exhibited 20 errors at session 1 maintained the improved level of performance observed at the end of the training at the recall session. All other drivers showed a large number of errors at the recall session (with two drivers showing more than 50 errors). As a key question associated with the driving performance of MCI drivers relates to the impact of the progression of cognitive decline on the performance, a longitudinal study allowing to understand how the performance degrades with the progression of cognitive decline is much needed. A large randomized controlled trial with a control MCI group exposed to the simulator without any feedback and healthy control groups (with and without feedback) would provide additional and important details about limitations and capabilities of MCI individuals when compared to healthy older drivers. Finally, participants in this study were a convenience sample of individuals with MCI. It would be important to determine if the smaller number of women that volunteered is indicative of a fear of being tested in this population.

In conclusion, this study shows that MCI individuals can be trained in a simulator to improve their driving. This improvement, however, appears to be labile for most MCI individuals that participated. This suggests, that regular rehearsal may be needed to maintain the improved performance. This decreased performance, however, was not beyond the performance observed at the first training session indicating that the MCI individual that were tested had maintained a safe, but not optimal driving performance during this period. Simulator training could be an important means not only for maintaining safe driving in MCI individuals, but also to offer cost-effective and safe means of evaluating how the progression of cognitive decline affects driving until it becomes unsafe.

## Author Contributions

All authors have fulfilled the criteria for authorship and have approved the final article.

## Conflict of Interest Statement

The authors declare that the research was conducted in the absence of any commercial or financial relationships that could be construed as a potential conflict of interest.
